# Practical Remarks on the Use of Digitalis in Consumption

**Published:** 1808-06

**Authors:** 


### *Practical Remarks on the Use of* DIGITALIS *in* CONSUMPTION. *By Dr.* SPENCE, *in a Letter to* * * * *.

SIR, *Dumfries, Dec.* 14, 1800.

I Shall submit to your consideration a short but candid view, divested as much as possible of technical terms, of my present mode of exhibiting the Digitalis in cases of pulmonary consumption; and in doing so, for the sake of perspicuity, shall divide the disease into two periods, the incipient and advanced stages, and add a few general observations.

1. In the incipient stage of consumption, when there is considerable vigour of constitution, particularly if attended with active hæmorrhagy from the lungs, I push the use of the digitalis cautiously, but freely; that is, I try to reduce the pulse under 60 strokes in a minute, and maintain this depression for two or three weeks, notwithstanding there may be occasionally considerable and distressing nausea. At the same time I advise a milk and vegetable diet, with gentle exercise on horseback, or in a carriage, when the weather will permit; and the use of the swing-chair, for an hour at a time, twice or thrice a day. When the pains about the chest are wandering, I also advise the repeated application of blisters, and other stimulating plasters, to the breast and between the shoulders; but if the pain be fixed, I prefer the introduction of a seton as near the part affected as possible. My patient is also directed to drink moderately of emollient teas or tar water, to be warmly clothed, to avoid cold and wet feet, and sitting up late at nights. All great exertions of the body, but particularly of the lungs, as singing or speaking aloud, must also be carefully avoided.

2. In the second or more advanced stage of the disease, accompanied with a quick pulse and great general debility, the treatment is very different. The fox-glove must be so managed as to lower the pulse and moderate the fever, but never pushed to such an extent as to excite nausea or sickness at stomach.

A little experience will soon enable a judicious and attentive practitioner to ascertain the doses adapted to his patient's constitution; and, as soon as he has attained this knowledge, he must be persevering in the use of the medicine. At this period of the disease the patient's strength must never be suffered to languish; he must be supported by nutritious diet. Agreeably to the present manners of society, two or three meals are taken in the day; but this mode of eating is very improper with delicate constitutions, more food being generally eaten at such stated periods than is necessary, thereby causing great heat, accelerating the pulse, and throwing the whole system into commotion. The diet should be nourishing, and of easy digestion, such as jellies, broths, eggs boiled soft, oysters raw or moderately roasted: indeed, a piece of fowl, beef, mutton, or venison, when attainable, dressed *rare*, may be taken in small quantities every two or three hours throughout the day. This deviation from the present fashion of eating is indispensible, ample nourishment being thereby thrown into the system without exciting irritation. At the same time that I recommend solid food in this way, I forbid the use of spices, wine, or spirits.

The same directions respecting local applications and exercise, are equally applicable to this as to the incipient stage, and particularly the exercise of swinging; and care must be taken that the swing chair be so constructed that the patient may be perfectly at ease, without being affected by fatigue or bodily exertions.

3. *Some general observations.* — Should a physician be anxious to predict the probable future effects of the fox-glove, I would request his attention to be particularly directed to the state of his patient's pulse. When there is a change from a hard and quick to a full and soft pulse, he may prognosticate flattering consequences.

I have, at present, patients taking this medicine in powder, tincture, and pill; but to the last form I now give a decided preference. The pill is generally most agreeable to the patient, and less apt to create nausea.

In only one instance have I found it bring on numbness of the extremities, stupor, great lassitude, and a reluctance to move. This happened to a young lady who was very far advanced in a consumption when she commenced the use of the fox-glove. When under this influence of the medicine, she had no cough, no pain, and her breathing was easy.

Dr. Adams, of Richmond, in this State, writes me, that a patient who was taking this medicine under my directions there, had his pulse reduced as low as 42, but then it beat irregularly.

Whenever these violent effects take place, the fox-glove must be omitted for two or three days, and then resumed in smaller doses.

It

It has only in one case proved laxative; but this was easily counteracted by combining it with opium, to the extent of half a grain or a grain in the course of twenty-four hours. I begin to think that these two medicines may be so combined and modified as to produce the finest effects in a variety of diseases, and particularly in the advanced stages of consumption; but as yet I have not had trials enough to authorise me to draw conclusions.

In most cases there has been a constipation of the bowels, which is easily as well as more safely removed by emollient injections than by cathartics. In such instances I recommended stewed prunes, and various preparations of rye meal.

In every case that has hitherto fallen under my notice, where the patient could bear the medicine well, I can assert that those colliquative or wasting sweats, so well known to physicians, and the constant and dreadful attendants of hectic fever, have invariably ceased as soon as the system was distinctly under the influence of the fox-glove.

In two cases of patients in the very last stage of the disease, who were extremely solicitous to make trial of the digitalis, I found the tinctnre, powder and pill, even in very moderate doses, create such a distressing nausea, and so completely destroy the appetite, that I was obliged to forbid its use. In one of these cases I thought a light watery infusion of columbo root, with a little orange peel, enabled the patient to bear the medicine somewhat better. It is therefore my present opinion, that no physician can pretend to form a justifiable prognosis, until he ascertains, on trial, how his patient's stomach will bear the medicine. My first patient, Drinan, fortunately experienced no uneasiness from large doses, excepting a temporary nausea; and I have now a young gentleman under my care, who lives about an hundred miles from this place, with whom the digitalis seems to agree extremely well. From his last letter, dated January 15, I shall give a short extract. And here it may be observed, that he was obliged to spend the last winter in the West Indies, on account of his pectoral complaints, and has now been taking the fox-glove more than twelve weeks under my direction.

"At present I think myself in a good way. My appetite not so ravenous; my digestion as well as can be wished; the pulse, for some time past, early in the morning, from 44 to 50, varying through the day from 70 to 90, and sometimes, when under exercise, to 100. My cough,

 in in the course of the twenty-four hours, not more than once or twice painful. The only drink I *indulge* in at present is a little tar water; but I am tired of it, and wish for a change."

The foregoing statement exhibits a general outline of my manner of administering the digitalis in consumptive cases, as well as the result of my present experience.—How far my opinions and practice may concur with the opinions and practice of others, I know not, having had no communications on the subject with any physicians, excepting with those who have almost implicitly followed my directions.

Before I close this letter, permit me to observe, that although I am, perhaps, the first person who announced in the American news-papers a case of phthisis pulmonalis successfully treated by the use of the digitalis, yet I am far from thinking it a certain cure in every case of pulmonary consumption. In the early stages of the disease, where there is no mal-conformation of body, I believe it will prove a successful remedy; but where this mal-conformation exists, I think it will not cure; neither do I think it will effect a cure in the very advanced periods of the disease. But even in the last and fatal stage, if the stomach will bear the medicine, it will mitigate distressing symptoms, and thereby tend to smooth the pangs of the declining patient.

We know the Peruvian bark will not cure every intermittent fever; but is the bark, therefore, to be undervalued? "Could we obtain," says an able writer, "a single auxiliary for the fox glove, such as we have, in many substances, for the bark, I should expect that not one case in five of consumption would terminate as ninety-nine in a hundred have hitherto done. But I believe a majority of cases will yield to simple fox-glove."

